# Two B or not two B; the question of bendamustine dosing in low grade lymphoma

**DOI:** 10.3389/pore.2025.1612195

**Published:** 2025-09-25

**Authors:** Eszter Földi, Ádám Wiedemann, Szabolcs Svorenj, Virág Réka Szita, András Dávid Tóth, Ilona Tárkányi, Ágnes Fehér, Ágnes Kárpáti, Laura Horváth, Gergely Szombath, Zsolt Nagy, Imre Bodó, Péter Farkas, Tamás Masszi, Gergely Varga

**Affiliations:** ^1^ Faculty of Medicine, Semmelweis University, Budapest, Hungary; ^2^ Department of Internal Medicine and Hematology, Semmelweis University, Budapest, Hungary

**Keywords:** follicular lymphoma, bendamustine, rituximab, schedule, low grade lymphoma

## Abstract

**Background:**

Follicular lymphoma (FL) is an indolent yet incurable B-cell lymphoma subtype commonly treated with a combination of bendamustine and anti-CD20 antibodies such as rituximab. While the standard administration involves a 2-day dosing schedule, the COVID-19 pandemic prompted the exploration of a 1-day regimen to reduce hospital visits for immunocompromised patients. This study aimed to compare the efficacy and safety of 1-day versus 2-day bendamustine regimens.

**Methods:**

We conducted a retrospective analysis of 144 patients with FL, marginal zone lymphoma, mantle cell lymphoma, or Waldenström macroglobulinemia treated at the Department of Internal Medicine and Hematology, Semmelweis University between 2015 and 2023. All patients received bendamustine combined with either rituximab or obinutuzumab. The primary endpoint was progression-free survival (PFS); secondary endpoints included overall survival (OS) and toxicity. Kaplan–Meier survival analysis and appropriate statistical tests were applied.

**Results:**

The median PFS for the cohort was 69.47 months; OS was not reached. Despite receiving a significantly lower cumulative dose, and being significantly older, patients on the 1-day regimen had similar PFS (not reached vs. 69.47 months; p = 0.885) and no significant difference in OS (p = 0.147) compared to the 2-day group. Adverse events were more frequent in the 2-day regimen group, including severe side effects, such as neutropenia (p = 0.044).

**Conclusion:**

A 1-day bendamustine regimen may offer comparable efficacy to the standard 2-day schedule, with a potentially more favorable toxicity profile and better convenience, especially in older or more vulnerable patient populations. These findings warrant further investigation in prospective randomized trials to establish optimal dosing strategies.

## Introduction

Follicular lymphoma (FL) is the second most frequently diagnosed lymphoma subtype, with an incidence of approximately 20 new cases per 100,000 individuals [1]. It primarily affects adults, with a higher incidence observed with increasing age. Although FL is classified as incurable, it is characterized by an indolent clinical course, and many patients remain asymptomatic following the time of diagnosis. Due to the slow progression of the disease patients can achieve a relatively long overall survival (OS) and a good quality of life with appropriate treatment. However, relapses are common, and approximately 20% of patients experience relapse within the first 2 years following initial therapy, complicating treatment strategies [2]. The management of FL involves a wide range of therapeutic options, including radiotherapy, chemotherapy, immunotherapy, targeted therapy, cellular therapy and their combinations. Still, chemotherapy is the most commonly used modality, with Bendamustine being one of the most widely utilized agents usually in combination with anti-CD20 antibodies, such as rituximab or obinutuzumab [2].

Bendamustine, an alkylating agent, was originally developed in the German Democratic Republic and has been in clinical use since 1969 for the treatment of multiple myeloma, chronic lymphocytic leukemia (CLL), non-Hodgkin lymphoma, and Hodgkin lymphoma. Although the drug was already available on the market for several years, formal clinical trials on Bendamustine did not occur until the 1990s. It was subsequently approved by the U.S. Food and Drug Administration (FDA) in 2008 for the treatment of CLL and indolent B-cell lymphomas [3]. In 2012, the combination of Rituximab and Bendamustine was proven to be non-inferior to the standard R-CVP/CHOP regimens as first line treatment for patients with indolent follicular and mantle-cell lymphomas. While no significant improvement in OS was observed, the combination therapy was associated with better PFS and a lower incidence of treatment-induced toxicities, rendering Rituximab-Bendamustine a favorable therapeutic option, therefore it has been utilized more and more in the last decades [4–7].

Bendamustine has also been evaluated in high-dose regimens as part of conditioning prior to autologous stem cell transplantation, but these approaches have been associated with considerable toxicity [8].

The Rituximab-Bendamustine combination is a preferred first-line treatment for classic follicular lymphomas with a high tumor burden, as well as for mantle cell lymphomas and marginal zone lymphomas. Rituximab-Bendamustine can also be used in the second-line setting if the regimen has not been previously used. Furthermore, Bendamustine is a second-line treatment option for diffuse large B-cell lymphomas in combination with Rituximab and Polatuzumab-vedotin. In Hungary its usage has been increasing gradually from 2015 onwards [4, 5, 9, 10].

Bendamustine reaches peak plasma concentrations approximately 1 hour after intravenous administration. It exhibits a relatively short elimination half-life and is metabolized via both hepatic and extrahepatic pathways. The resulting metabolites are primarily excreted in the urine, with a smaller proportion eliminated via the feces. Notably, clinically significant accumulation does not occur with standard dosing [11].

The standard administration of Bendamustine involves four to six cycles of intravenous infusion at a daily dose of 90 mg/m^2^ over two consecutive days combined with 375 mg/m^2^ rituximab on day 1, with appropriate premedication to mitigate adverse effects. However, during the COVID-19 pandemic, minimizing patient contact became a priority for immunosuppressed individuals. As a result, in many hospitals incuding ours, a modified dosing regimen was implemented, wherein patients received a single-day dose of Bendamustine instead of the standard 2-day protocol. This modification raised important questions about the potential impact on treatment outcomes, particularly in terms of progression-free survival (PFS) and overall survival.

The objective of this study is to investigate whether the modified 1-day regimen of Bendamustine significantly impacts PFS and OS compared to the standard 2-day regimen. This analysis aims to provide insights into the feasibility of adopting this adjusted dosing schedule without compromising the efficacy of treatment.

## Materials and methods

### Patients

We retrospectively collected data from 144 patients treated at the Department of Hematology and Internal Medicine, Semmelweis University, between 2015 and 2023. Eligible patients had a histologically confirmed diagnosis of one of the following: follicular lymphoma, marginal zone lymphoma, mantle cell lymphoma, or Waldenström macroglobulinemia established according to the WHO diagnostic criteria of the current era [12]. All selected patients received bendamustine in combination with either rituximab or obinutuzumab. Adverse events were collected from the electronic casenotes manually. The study was approved by the Hungarian National Ethics Committee, the patients provided full informed consent.

### Statistical analysis

The primary endpoint was PFS, defined as the time from treatment initiation to disease progression or death. Follow up was typically performed every 3 months from treatment completion, and involved physical examination and laboratory tests, with imaging at the suspicion of progression. OS was evaluated as a secondary endpoint, we contacted all patients to confirm OS status. We also aimed at evaluating retrospectively the toxic side effects in the two groups based on laboratory data and casenotes.

Comparisons of categorical variables were performed using Fisher’s exact test, and continuous variables were analyzed using the Mann–Whitney U test. Survival analyses for PFS and OS were conducted using the Kaplan–Meier method. All statistical analyses were performed using SPSS version 29.0.1.0 (IBM Corp., Armonk, NY, United States).

The study was approved by the Institutional Review Board and informed consent was obtained in accordance with the Declaration od Helsinki.

## Results

In the overall study population, the PFS was 69.47 months, while OS had not yet been reached at the time of analysis. The majority of patients were diagnosed with low-grade follicular lymphoma or marginal zone lymphoma (64.3%) and received bendamustine as first-line therapy (84.7%), typically in combination with rituximab (87.5%). The majority of patients (89.5%) received four to six cycles of Bendamustine. Fewer cycles were administered only in cases of severe allergic reactions or when the patient’s general condition did not allow the continuation of treatment. Approximately half of the patients received maintenance rituximab (Table 1).

**TABLE 1:** Patients’ characteristics.

Patients characterisctics		All patients	2-day regimen	1-day regimen	p
No. of patients		144	103	41	
Mean age	at diagnosis	66.19	64.47	70.50	0.008
	at treatment	67.53	65.86	71.72	0.009
Histological diagnosis	FL1-2	47 (32.6%)	39 (37.9%)	8 (19.5%)	0.037
	FL3a	13 (9.0%)	10 (9.7%)	3 (7.3%)	
	MZL	60 (41.7%)	41 (39.8%)	19 (46.3%)	
	MCL	7 (4.9)	2 (1.9%)	5 (12.2%)	
	W	17 (11.8%)	11 (10.7%)	6 (14.6%)	
Line of treatment	1	122 (84.7%)	90 (87.4%)	32 (78.0%)	0.404
	2	17 (11.8%)	9 (8.7%)	8 (19.5%)	
	3+	5 (3.5%)	4 (3.9%)	1 (2.5%)	
anti-CD20	Obinutuzumab	18 (12.5%)	17 (16.5%)	1 (2.4%)	0.024
	Rituximab	126 (87.5%)	86 (83.5%)	40 (97.6%)	
Maintenance	Received	71 (49.3%)	55 (53.4%)	16 (39.0%)	0.141
	Not received	73 (50.7%)	48 (46.6%)	25 (61.0%)	

FL1-2, Follicular lymphoma grade 1-2; FL3a, Follicular lymphoma grade 3a; MZL, Marginal zone lymphoma; MCL, Mantle cell lymphoma; W, Waldenström macroglobulinaemia.

### Baseline parameters

There were no statistically significant differences between the two treatment groups regarding gender, line of therapy, number of treatment cycles, or the type of anti-CD20 antibody used (rituximab vs. obinutuzumab). However, a significant difference in mean age was observed between patients receiving the 2-day regimen (median = 68.45 years) and those on the 1-day regimen (median = 74.83 years; p = 0.009).

Analysis of baseline laboratory parameters revealed significant differences in platelet count and lactate dehydrogenase levels, both of which were higher in the 1-day regimen group (p = 0.028 and p = 0.022, respectively). No significant differences were observed in other parameters collected, such as hemoglobin level, neutrophil count, or total leukocyte count between the groups.

### Survival

**FIGURE 1:**
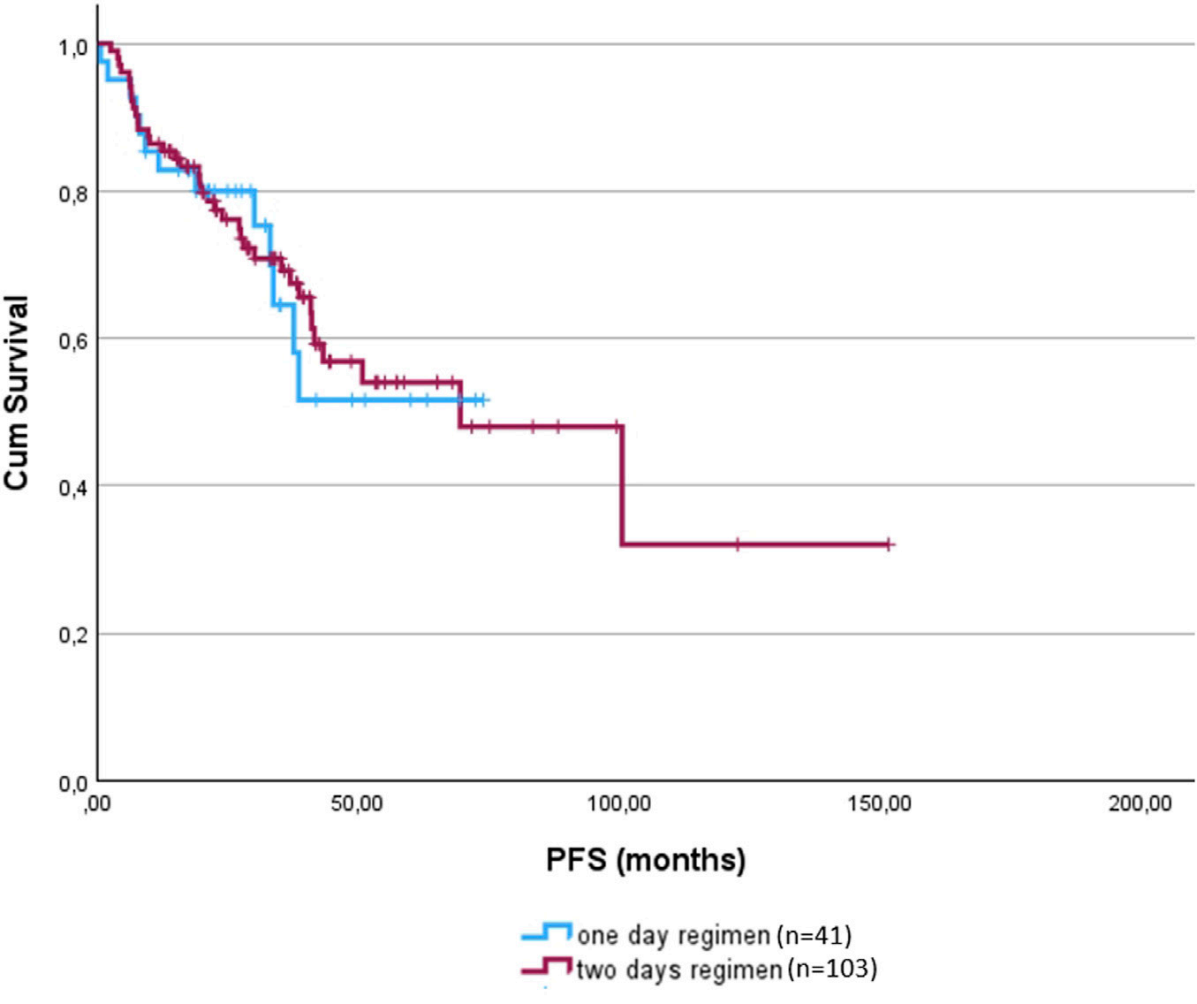
Progression free survival of the 1-day (blue) and 2-day regimens (red).

The total dose was adjusted for body surface area to allow for more objective comparisons. The 1-day regimen was associated with a significantly lower cumulative dose of bendamustine (p < 0.001). The mean total dose delivered in the 1-day regimen group was 393.21 mg/m^2^ (range: 50–800 mg/m^2^), compared to 694.17 mg/m^2^ (range: 100–1125 mg/m^2^) in the 2-day group. Importantly, despite this difference in dosing and age, there was no significant difference in PFS between the two groups (1-day: not reached; 2-day: 69.47 months; p = 0.885). OS had not been reached in either group, with no significant difference observed (p = 0.147) (Figure 1).

Adverse events were reported in both groups (Table 2) and were more frequent in the 2-day regimen group (p = 0.077). Notably, all cases of neutropenia (n = 7) occurred in the 2-day group, which was proven to be statistically significant (p = 0.044).

**TABLE 2:** Adverse events in the one and two days groups.

Adverse events	All patients	2-day regimen	1-day regimen
fever	13	9	4
rash	18	13	5
headache	1	0	1
fatigue	6	5	1
collapsus	1	1	0
dizziness	2	2	0
nausea	13	10	3
diarrhea	5	2	3
neutropenia	7	7	0
tumorlysis syndrome	2	1	1
bone marrow depression	1	1	0
infusion reaction	4	4	0
toxicoderma	2	2	0

Our analyis was not powered to perform formal noninferiority tests.

**FIGURE 2:**
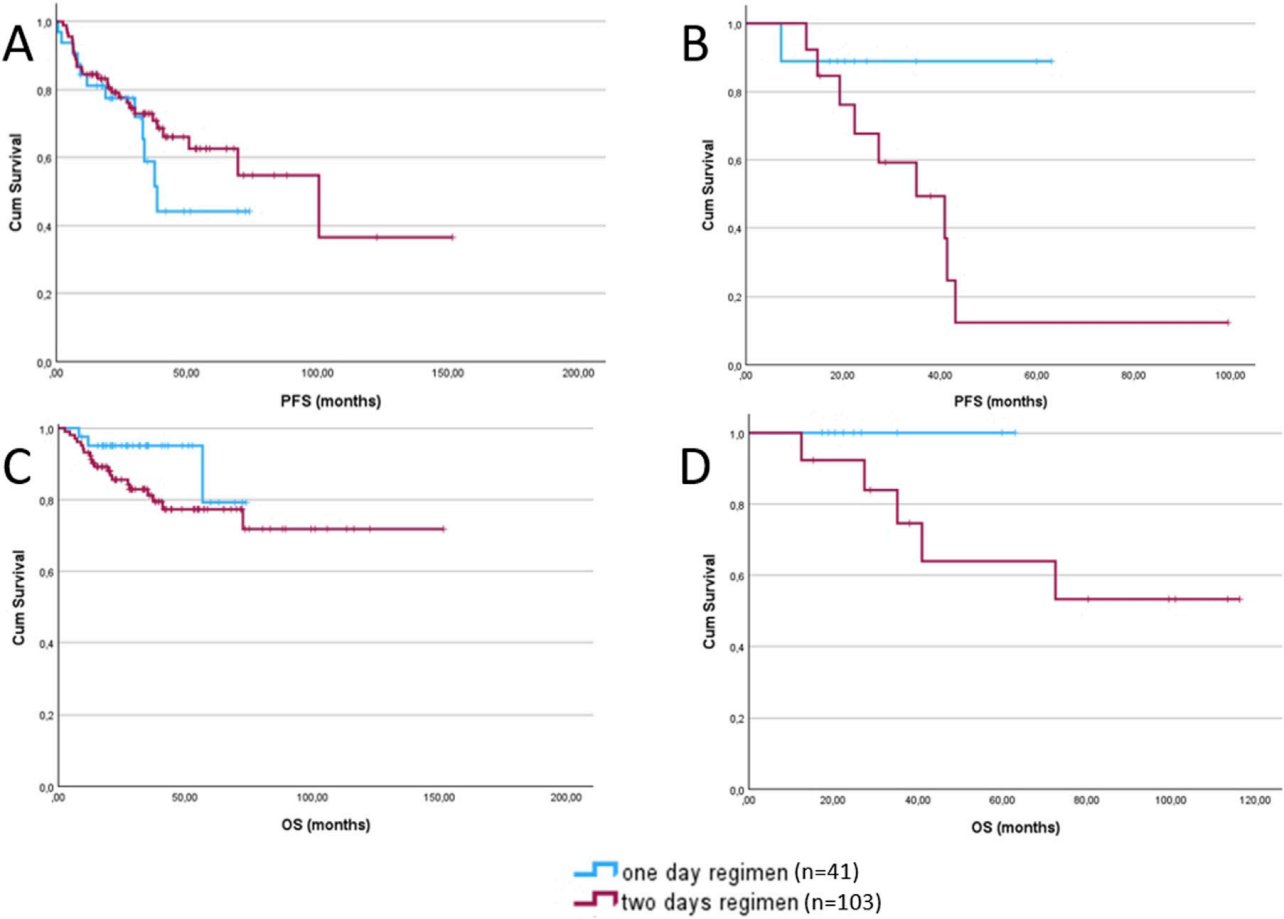
Progression-free survival **(A)** and overall survival **(C)** of patients receiving bendamustine as first-line treatment, comparing the 1-day regimen (blue) and the 2-day regimen (red). Progression-free survival **(B)** and overall survival **(D)** of patients receiving bendamustine as second or subsequent lines of treatment, comparing the 1-day regimen (blue) and the 2-day regimen (red).

To enable a more objective evaluation of our results, we analyzed first-line and subsequent lines of therapy separately as well. Among patients who received Bendamustine as first-line therapy, no significant differences were observed in PFS or OS (Figures 2A,C) between dosing regimens (p = 0.885, p = 0.149). As the majority of patients belonged to this subgroup, the results closely reflected those of the overall cohort.

In contrast, among those treated with Bendamustine in the second-line or later setting, although differences in PFS and OS (Figures 2B,D) did not reach statistical significance, patients receiving the 1-day dosing regimen demonstrated a trend toward improved outcomes in both PFS and OS (p = 0.081, p = 0.230).

## Discussion

This retrospective analysis aimed to evaluate the clinical efficacy of a 1-day versus 2-day bendamustine regimen in patients with indolent B-cell lymphomas. Most of the patients in the 1-day group had their treatment during and following the COVID pandemy, despite this and the significantly lower cumulative dose of bendamustine administered in this group, PFS did not differ significantly between the groups, suggesting that the altered dosing schedule may be non-inferior in terms of remission rates.

The lack of a significant difference in OS, which had not yet been reached in either group at the time of analysis, further supports the potential viability of the 1-day regimen as a more convenient and possibly equally effective alternative. However, longer follow-up is warranted to confirm this observation.

Importantly, the incidence of adverse events, particularly neutropenia, was higher in the 2-day regimen group. All documented cases of neutropenia occurred exclusively in this group, suggesting that the standard regimen may carry a higher toxicity risk. While the overall adverse event rate difference did not reach statistical significance, the trend favors the shorter regimen in terms of tolerability.

The findings of this study are consistent with those reported by Masamoto, Shimura, and Kurokawa (2022), who also employed a reduced 1-day bendamustine dosing regimen (two-thirds of the standard dose) in elderly patients with follicular lymphoma. Similar to the present study, they observed no significant difference in PFS between the standard and reduced dosing groups [13]. However, the retrospective nature of this study, the relatively small sample size, and potential selection bias (e.g., older patients more often receiving the 1-day regimen) limit the generalizability of the conclusions.

Prospective, randomized controlled trials would be required to validate these findings and to better elucidate the optimal dosing schedule for bendamustine, especially in elderly or comorbid populations where treatment-related toxicity is a critical concern.

In recent years, the therapeutic landscape for indolent B-cell lymphomas has continued to evolve with the development of novel agents, including next-generation alkylating agents such as brextamustine. Brextamustine has shown promise in early-phase trials, offering potential improvements in efficacy and tolerability [14].

Another frontline option is rituximab-lenalidomid often mentioned as R2 protocol, which offers patients a chemotherapy free approach with compatible efficacy [15]. The side effect profile of this protocol is different from that of rituximab-bendamustine, but marrow suppression and coagulopathy remain significant issues. In the relapsed setting there are numerous new candidates including CAR-T cells, T-cell engagers, antibody-drug conjugates, EZH2 inhibitors and at least some of these options will soon enter the front line field [16].

However, despite these advances, rituximab in combination with bendamustine remains a widely used frontline regimen. This preference is largely driven by the regimen’s well-established efficacy, manageable toxicity profile, and significantly lower cost compared to the emerging therapies. In healthcare systems where cost-effectiveness plays a major role in treatment decisions, rituximab-bendamustine continues to be a effective and accessible option for most patients.

## Data Availability

The data analyzed in this study is subject to the following licenses/restrictions: The original contributions presented in the study are included in the article/supplementary material, further inquiries can be directed to the corresponding author. Requests to access these datasets should be directed to vargager@gmail.com.
